# Breast Tumor Tissue Segmentation with Area-Based Annotation Using Convolutional Neural Network

**DOI:** 10.3390/diagnostics12092161

**Published:** 2022-09-06

**Authors:** Bendegúz H. Zováthi, Réka Mohácsi, Attila Marcell Szász, György Cserey

**Affiliations:** 1 Faculty of Information Technology and Bionics, Pázmány Péter Catholic University, 1083 Budapest, Hungary; 2 Department of Internal Medicine and Oncology, Semmelweis University, 1083 Budapest, Hungary; 3 2nd Department of Pathology, Semmelweis University, 1091 Budapest, Hungary

**Keywords:** breast cancer, medical image classification, histopathological image segmentation, whole slide image analysis, deep learning, convolutional neural networks, sliding window method, computer-aided diagnosis

## Abstract

In this paper, we propose a novel approach to segment tumor and normal regions in human breast tissues. Cancer is the second most common cause of death in our society; every eighth woman will be diagnosed with breast cancer in her life. Histological diagnosis is key in the process where oncotherapy is administered. Due to the time-consuming analysis and the lack of specialists alike, obtaining a timely diagnosis is often a difficult process in healthcare institutions, so there is an urgent need for improvement in diagnostics. To reduce costs and speed up the process, an automated algorithm could aid routine diagnostics. We propose an area-based annotation approach generalized by a new rule template to accurately solve high-resolution biological segmentation tasks in a time-efficient way. These algorithm and implementation rules provide an alternative solution for pathologists to make decisions as accurate as manually. This research is based on an individual database from Semmelweis University, containing 291 high-resolution, bright field microscopy breast tumor tissue images. A total of 70% of the 128 × 128-pixel resolution images (206,174 patches) were used for training a convolutional neural network to learn the features of normal and tumor tissue samples. The evaluation of the small regions results in high-resolution histopathological image segmentation; the optimal parameters were calculated on the validation dataset (29 images, 10%), considering the accuracy and time factor as well. The algorithm was tested on the test dataset (61 images, 20%), reaching a 99.10% f1 score on pixel level evaluation within 3 min on average. Besides the quantitative analyses, the system’s accuracy was measured qualitatively by a histopathologist, who confirmed that the algorithm was also accurate in regions not annotated before.

## 1. Introduction

Malignant tumors are one of the leading causes of death in the world’s population. Among all types of cancer, breast cancer is the most common malignant disease in women. According to statistics, every eighth woman will be diagnosed with breast cancer in her life. There is an unforeseen increase predicted in the EU regarding new cancer cases in men by 2040 [1]. Women, although less dramatically, will also witness a rise in tumor numbers. Cancer incidence is expected to grow by 30%, and cancer-related death will increase by 35% in Hungary by 2030 [2]. Therefore, there is an urgent need for improvement in diagnostic capabilities and early detection of cancerous diseases. The diagnosis of a breast tumor is based on physical examination, imaging and histological confirmation. Shortage in any of the above resources will hinder cure and outcome. As the analyses are time-consuming and experts are overloaded, obtaining a diagnosis quickly and reliably is often a difficult process in healthcare institutions, thus, the treatment of the patient may be delayed. In the future, pathologists will perform histological analyses with tools not utilized previously, quality assurance, computer-aided assistance, digitalization and accuracy of measurements will reach the level of “next-generation histology”.

**Figure 1:**
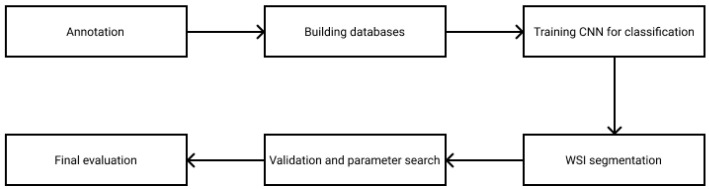
Flowchart of the research process.

In this paper, a new rule-based segmentation technique is introduced through breast tumor tissue evaluation. The flowchart in Figure 1 presents how the research was managed. The WSI annotation was executed by the experts on digitized breast tissue surgical slides. After preprocessing the data and splitting it into training, validation and test sets, small ROIs were created from the marked areas. As the database was prepared, a convolutional neural network was trained to classify the patches into the tumor or cancer types. The segmentation strategy combines the ROI classification with a sliding window algorithm, where the essential parameters were optimized on the validation dataset. Finally, the algorithm was evaluated on the test set, reaching a 99.10% f1 score. This paper is designed to provide a deeper understanding of the applied rules, which could lead to accurate results on other medical computer vision problems as well.

## 2. Materials and Methods

### 2.1. Algorithm and Implementation Rules

Facing segmentation problems on large-scale images is a complex task, especially from a medical point of view where it is essential to precisely segment regions and to avoid false negative results. Following the rules written in this paper, we provide a template to accurately solve high-resolution biological segmentation tasks in a time-efficient way regardless of the imaging technique. Our strategy reflects a new aspect of a tumor tissue segmentation algorithm, from the data annotation to the evaluation. The following rules were applied and can be applied efficiently in other segmentation tools or in other tissue types:

1. **Database rule: avoid human errors in the annotation**
  In general, analyzing cancer tissues generates contour-based annotations for the whole image, separating the tumor and healthy parts by a border. Due to our special request, the experts annotated the samples for this research in a very time-consuming way by marking only the tumor and healthy areas without any intermediate parts. This extra effort results in a more precise database with less noise, human error and redundant data compared to other datasets.
2. **Preprocessing and evaluation rule: involving the experts**
  For medical image processing, it is essential to follow the instructions of the experts, so during the study, they were involved in the research at every stage of the implementation. Setting the size of the training tiles was examined by the histopathologists to select the one that is descriptive enough for the human eye. The segmentation results were also evaluated qualitatively by the experts with multiple parameter settings.
3. **Training rule: multi-resolution database**
  In the case of deep learning tasks, it is crucial to building a model that is generalizable to different datasets. Considering the fact that there is no optimal measure for the best resolution where the most information can be extracted, for neural network training, we used a multi-resolution collection of the tumor and healthy tiles to be more robust to noise and magnitude changes.
4. **Validation rule: time–accuracy trade-off**
  The aim of our study was to create an accurate algorithm to segment tumor and healthy tissues depending on the evaluation time for computer-aided diagnosis. This algorithm used hardware parallelization, time–accuracy trade-off measurements on the sliding window classification and appropriate parameter optimization to speed up the segmentation process.

### 2.2. Database and Preprocessing

Our database includes high-resolution, H&E stained bright field microscopic histopathological images collected by Semmelweis University. The samples are collected from breast tissue surgical resections.

In most cases, researchers use histological slices from formalin-fixed, paraffin-embedded (FFPE) tissue samples, which will be stained with hematoxylin and eosin. Obtaining patient samples is easy through the various clinics. The prepared slices are examined by the pathologist under a light microscope at different magnifications to make an accurate diagnosis, and, in many cases, the slices are stained for other examinations. In routine diagnostics, a slide can be examined within seconds to minutes, and no slides are digitized. There are many scanners that already have state-of-the-art technology. Electronic slices are made by WSI scanners, which allow reading the slices and storing the images on the one hand and using programs to display the electronic slices (samples/images) on the other hand.

**Figure 2:**
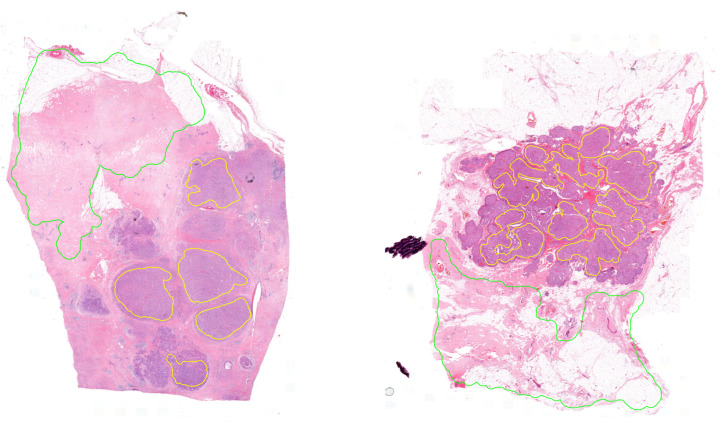
Examples of annotated histopathology images. Yellow annotation: tumor, green annotation: normal tissue.

The database contains 291 high-resolution 8-bit depth RGB images in 20× magnification, which has 254,632 ±34,643× 177,585 ±83,086 pixel size on average (Figure 2). The pixel width is 0.2425, and the pixel height is 0.2426 micrometers. These images were annotated in the open-source QuPath software environment by pathologists marking the surely healthy and tumor regions. The experts were asked to annotate the samples to our request in a special way by annotating only the tumor and healthy regions without any intermediate parts (Rule 1). This process is very time consuming and requires more effort from the histopathologists because they marked the separated cancerous regions independently (area-based annotation) instead of simply drawing a border between the tumor and normal regions (contour-based annotation). Therefore, not all the regions were annotated. Thus the aim of this study was to find all the cancerous regions by learning the features of the tissues. Using area-based annotation helps to create a more precise dataset containing less noise, human error and redundant data.

**Figure 3:**
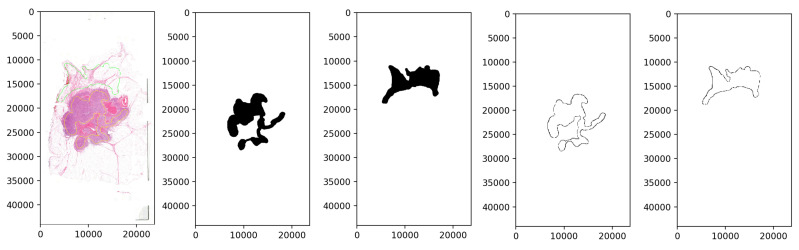
The process of masking the annotated areas (yellow: tumor, green: normal) and cutting out the inside patches of histopathological image regions.

**Figure 4:**
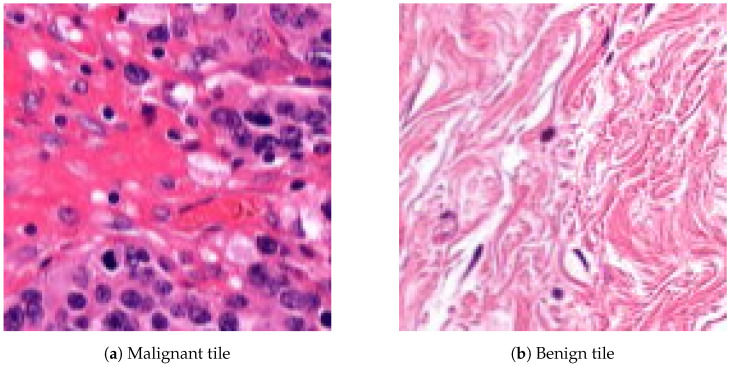
Examples of 128 × 128-pixel resolution annotated histopathological image patches.

The database preprocessing was implemented as follows. First, we built a Groovy script as a QuPath Plugin, which masked the white background from the samples to decrease the size and keep only the relevant data. Due to the big size of the images, a downsampling method was applied. Thus the samples were exported through lossless compression from QuPath data files to *.tiff* format with 4-6-8 downsampling factors to make a multi-resolution database (Rule 3), which helps the network evaluation to be robust and flexible at different magnifications. The second step, the annotation masking and the image patch extraction, was executed in Python (Figure 3). Ground truth is needed to evaluate the algorithms, so generating arrays to store the healthy and tumor masks helps us measure the accuracy of the analysis. Small, 128 × 128-pixel resolution tiles (Figure 4) were cut from the regions that the expert considered surely as tumor or as normal tissue areas (Rule 2). This size was small enough to observe just a few cells precisely and large enough to notice some local correlations in the tissues.

This database was equally distributed by the downsampling factor, and it was split into three groups: 70% (201 images) belong to training, 10% (29 images) to validation and 20% (61 images) to the testing dataset (Table 1).

These database samples were, on average, 29,314 ± 10,908 pixels wide and 17,432 ± 5126 pixels long. The training database contains 206,174 disjunct patches, the validation database 30,927 patches and the test database 36,944 patches cut out from the annotated image regions. At the neural network training and at all the measures and evaluations, the healthy-tumor tile rate was normalized and equalized.

### 2.3. Training and Feature Extraction

The hardware used in this research is the NVIDIA RTX 2080 GPU [3]. The neural network was trained in the TensorFlow Keras framework (version 2.8.0). The algorithms were implemented by scripts written in Python and evaluated by the scikit-learn machine learning library, such as measuring the accuracy, f1 score, precision, recall, area under the ROC curve (auroc), mean squared error and confusion matrix scores.

**Figure 5:**
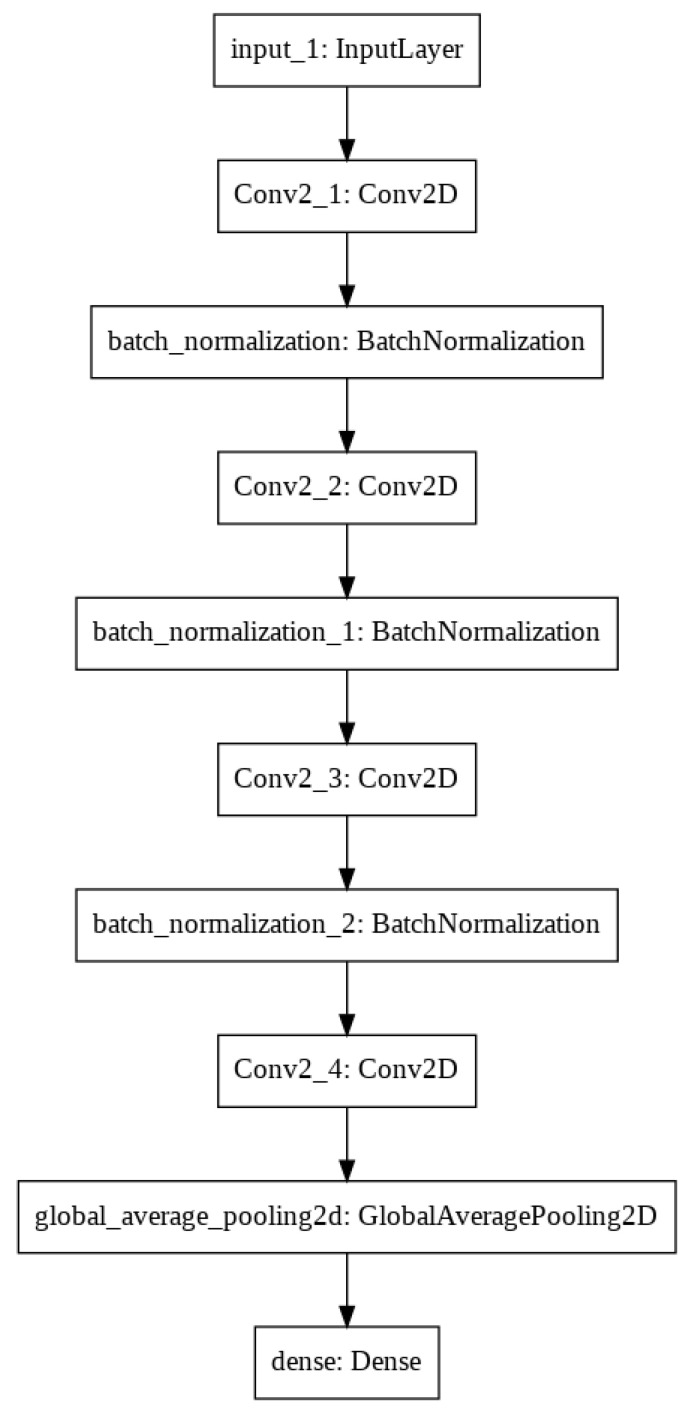
Convolutional neural network model.

**Figure 6:**
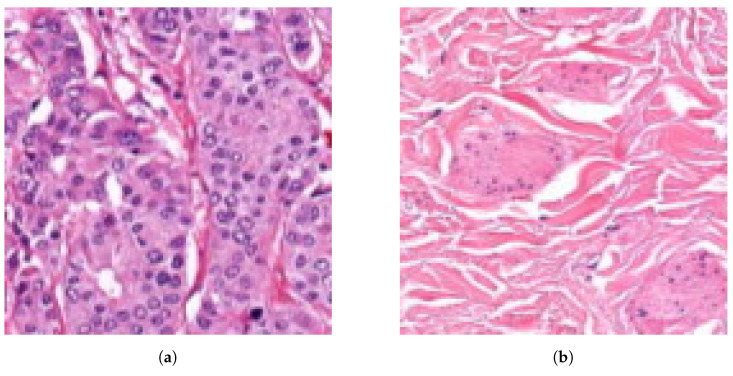
Examples of 128 × 128-pixel resolution histopathological image tile evaluations. (**a**) Image tile prediction: tumor confidence = 0.99 (tumor). (**b**) Image tile prediction: tumor confidence = 0.01 (normal).

Training a neural network is a very costly process at the beginning without any previous knowledge, so we made a proof of concept of a small set of breast tissue samples, which enabled us to perform more experiments in a shorter time. Initially, we started the training with a deep neural network to achieve high test accuracy according to a large number of network parameters. After that, we reduced the filter sizes to be as small as possible, which still keeps the training accuracy high. After testing a lot of convolution neural network parameters and filter sizes, the final architecture has four convolutional layers with filter sizes of 8, 16, 16 and 32 and convolutional kernel sizes of 3 × 3, 3 × 3, 5 × 5 and 3 × 3 (Figure 5). After we found approximately good architecture and hyperparameters resulting in accurate classification scores, we expanded the database for all breast tumor tissue samples using 90% (185,874 patches) of the 128 × 128 resolution tiles from the training dataset weighted by the rate of cancerous and healthy tissue patches. The patches were added to the neural network in 64 large batches, which was efficient considering the memory usage and the training performance. After many attempts to avoid underfitting and overfitting problems by making small changes in the hyperparameter settings—such as modifying the learning rate, and increasing the bath size—the final neural network architecture provides 64,290 parameters. After each layer, the SELU activation function was applied to avoid the dying ReLU problem, and a batch normalization was added to optimize the training performance. The last layer was a fully connected dense layer with a softmax activation function, which returns as linear regression confidence between 0 and 1 (0 means healthy and 1 means tumor). The final decision was made according to 130 combined parameters, and the training threshold between the two classes was at a 0.5 confidence value (Figure 6).

For training the neural network, the SGD optimizer was used with a momentum of 0.75 and a learning rate of 1e-4, which helps the convergence become faster. The weights were L2-normalized for affecting the network to be based on distributed decisions instead of being dominated by a few big weights. The back-propagation starts calculating from the mean squared error scores. The training performance was evaluated on 10% (20,300 patches) of the training dataset. The model keeps learning until the validation loss decreases, and early stopping is applied to avoid getting stuck at the local minimum. The best weights were saved at the minimum point of the validation loss.

**Figure 7:**
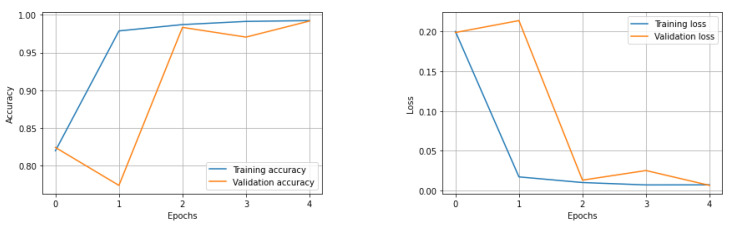
Training and validation loss accuracy changes at every epoch during training.

**Figure 8:**
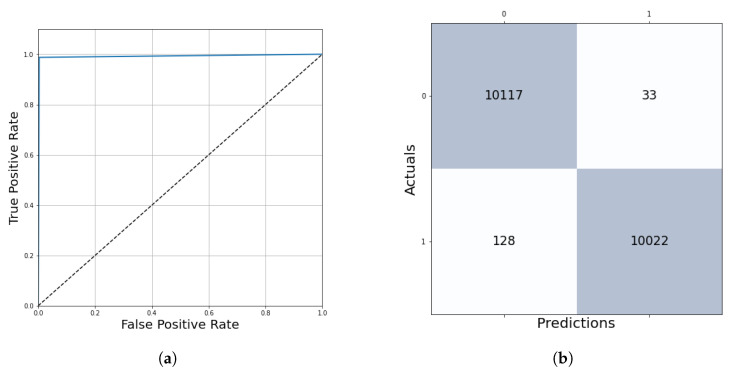
ROC curve and confusion matrix to evaluate the training performance. (**a**) Averaged ROC curve. (**b**) Confusion matrix.

After five epochs of training, the network stopped learning with a training loss of 0.79% and a validation loss of 0.59% (Figure 7). The performance evaluation on 10% of the training dataset shows us the following results: accuracy = 99.21%, auroc = 99.20%, precision = 99.67%, recall = 99.21%, f1 score = 99.20%. The True Positive region of the confusion matrix is 99.67%, the True Negative region is 98.74% and the False Positive and False Negative regions are 0.33% and 1.26%, respectively (Figure 8). This network learned the required features in order to be able to precisely and confidently classify the 128 × 128 resolution unknown patches into tumor and healthy groups.

### 2.4. Whole Slide Image Segmentation Strategy

In the previous section, the deep learning algorithm was described, which is an evaluation of a 128 × 128-pixel-sized patch of tissue, and the returned value is the result of the linear regression between 0 (healthy) and 1 (tumor). The algorithm learned the parameters of the cancerous and healthy tissues, so it is also able to predict unknown patches by their features.

The algorithm segments the whole slide of histopathological images by a sliding window technique, which steps through the whole image by evaluating 128 × 128-pixel-sized ROIs (Rule 4). There are parameters that have to be optimized to get an accurate and time-efficient algorithm. First, the ideal threshold level should be chosen between 0 and 1, which determines the boundary between the healthy and tumor decision.

The second parameter that should be optimized is the step size, which sets the level of overlapping during the sliding window algorithm. Trying 128 (no overlapping), 64, 32, 16 and 8 step sizes were the logical options considering the hardware characteristics. Evaluating just one tile takes 0.019 s, but appropriate hardware parallelization is recommended to speed up the process. With batch-level evaluation, the algorithms were significantly faster, so this is an efficient way to develop a real-time calculating assistant program. However, the difference of the step sizes was important because there are, on average, 31,190 evaluations at step size = 128, 124,758 at 64, 499,034 at 32, 1,996,139 at 16 and 7,984,558 at 8. Decreasing the step sizes leads to twice as many evaluations in both the horizontal and vertical directions. After evaluating each patch, the predictions should be aggregated at every pixel. Therefore, each pixel got a different tumor confidence value calculated from 1 prediction at step size = 128, 4 predictions at step size = 64, 16 predictions at step size = 32, 64 predictions at step size = 16 and 256 predictions at step size = 8. Displaying the whole slide image, evaluations were implemented as follows: every prediction value becomes a color, which shows the tumor confidence of that area. The selected palette was grayscale (from 0 = white to 1 = black), and the Jet colormap from Matplotlib external library in Python represents the values from blue to red (0 to 1). The performance evaluation of these algorithms is based on the binarized values at each pixel that were compared with the ground truth markers by the pathologist experts.

Due to the long evaluation time and the changing accuracy depending on the assigned values for each pixel, a parameter search was needed to find the best setups for this algorithm to evaluate whole slide images precisely and quickly. These optimization algorithms were executed on the validation dataset written in the following section.

### 2.5. Validation and Parameter Optimization

**Figure 9:**
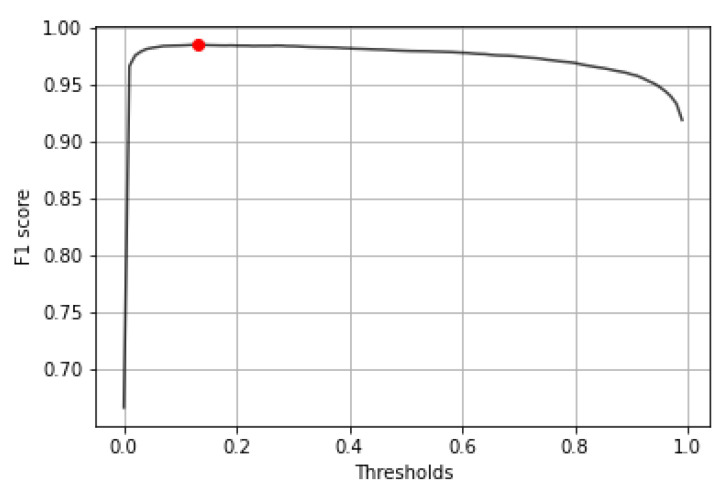
Displaying f1 score changes at different threshold levels between 0 and 1.

The parameter searching was executed on the validation dataset, which is 10% of the database, containing 29 high-resolution samples having 30,927 validation patches. The first step was finding the best threshold to binary classify the results of the output confidence. After trying all the thresholds between 0.00 and 1.00, the final threshold with the highest f1 score was 0.13 (Figure 9). The kernel level evaluation on 30,927 validation patches at this threshold shows us the following results: accuracy = 98.52%, auroc = 98.52%, precision = 98.22%, recall = 98.84%, f1 score = 98.53% and mean squared error = 1.48%. The True Positive region of the confusion matrix is 98.21%, the True Negative region is 98.84% and the False Positive and False Negative regions are 1.79% and 1.16%, respectively.

**Figure 10:**
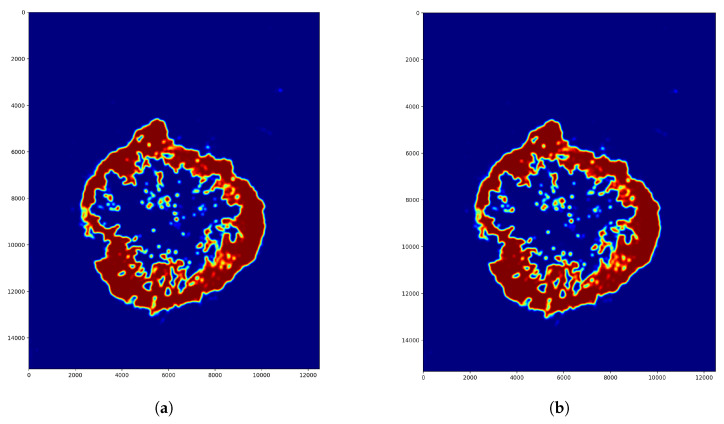
Parameter searching for the optimal overlapping step size. (**a**) Step size = 32 pixels. Pixel value averaged from 16 predictions. (**b**) Step size = 8 pixels. Pixel value averaged from 256 predictions.

**Figure 11:**
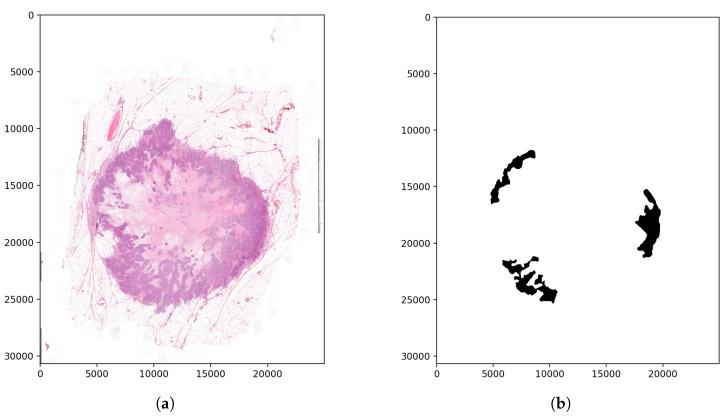
Examined tissue image and the tumor annotations. (**a**) Image of the examined tissue sample. (**b**) Annotation mask of the tumor regions.

**Figure 12:**
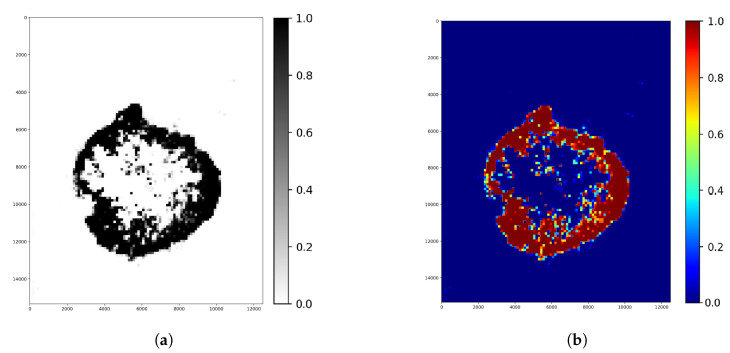
Whole image (Figure 11a) segmentation displays the evaluation before optimizing the parameters. In this case, there is no overlapping, so the pixel value came from a one-tile prediction of each tile. Colors around the 0 confidence value means healthy, and around 1 means tumor tissue region. (**a**) Segmentation result (Binary colormap). (**b**) Segmentation result (Jet colormap).

**Figure 13:**
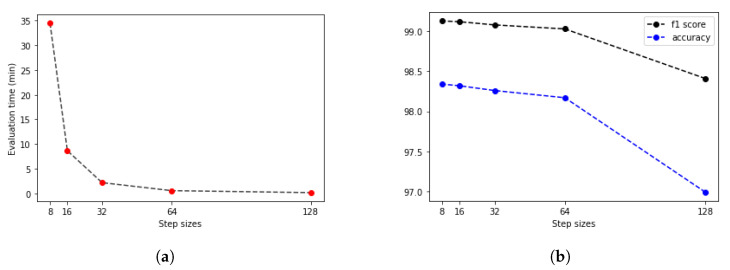
Parameter searching and validation to find the best step size considering time and accuracy. (**a**) Evaluation time depending on step sizes. (**b**) F1 score and accuracy changes.

The second step was finding the ideal step size depending on time and f1 score. For this parameter, searching the samples was evaluated at different step sizes with the final threshold level (0.13). The validation dataset samples were evaluated with the previous 128, 64, 32, 16 and 8 pixels step sizes (Figure 10). The accuracy of these algorithms was calculated by comparing the annotated masks as the ground truth (Figure 11b) with the predictions (Figure 12) at each pixel position. The time of the evaluation shows us exponential growth, so the goal is to use as few evaluations as possible to become accurate (Figure 13). Beyond step size 32, the algorithm’s accuracy is not increasing significantly, but the time changes are rising. At the step size parameter of 32 pixels, the algorithm is averaging the returning values from 16 predictions. Using more overlapping at the sliding windows causes such a large amount of time that makes the algorithm become inefficient for real-time diagnosis. The final evaluation time takes 2.15 min plus the assigning time of the predictions (0.31 min), so the total time of a sample evaluation is executed on average just within 3 min (Table 2).

**Figure 14:**
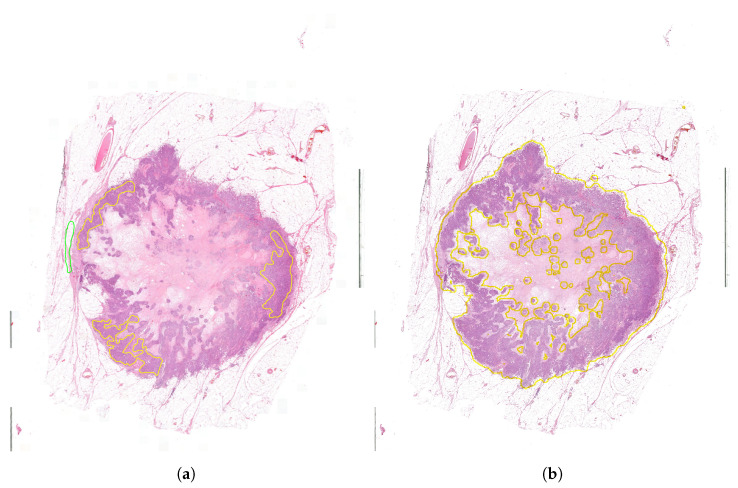
Whole slide image segmentation results with threshold = 0.13 and step size = 32 pixels. (**a**) Ground truth annotations (yellow: tumor, green: normal/healthy). (**b**) Segmentation result delineated onto the original image (yellow: tumor, rest: normal/healthy).

Considering the results of the validation algorithms, the final parameter for the decision threshold between healthy or tumor tissue tiles was 0.13 (Figure 9). The step size for the sliding window evaluation was 32 pixels (Figure 10b), which means 16 for prediction overlapping. The results are displayed on Figure 14.

## 3. Results

### 3.1. Kernel Level Evaluation

**Figure 15:**
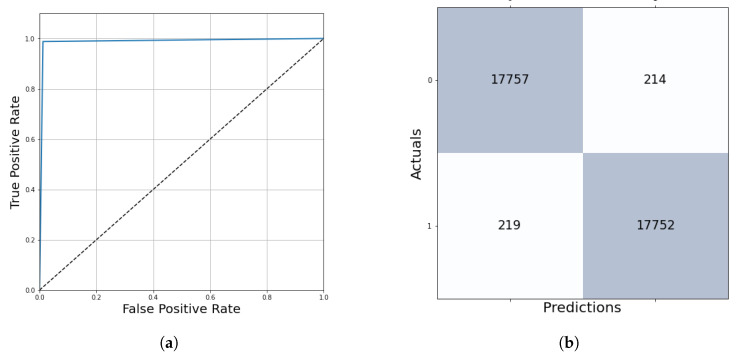
ROC curve and confusion matrix evaluation on the test dataset. (**a**) Averaged ROC curve. (**b**) Confusion matrix.

The algorithm’s performance was evaluated on the test dataset containing 61 multi-resolution whole-slide images, which is 20% of the samples in the database. The results were measured in two ways. At kernel level evaluation—based on the prediction of 36,944 test patches—ensures **98.80%** accurate outcomes with the final threshold of 0.13 (Table 3, Figure 15).

### 3.2. Pixel-Level Evaluation

The second way was the pixel-level evaluation, where all the pixels of the ground truth annotations were compared to the whole slide image predictions at the same positions. The quantitative evaluation—with the final threshold of 0.13 and with a 32-pixels step size—shows us that the algorithm at this parameter setting provides quick and accurate predictions about the ground truth areas. The averaged pixel level accuracy was **98.28**%, and the f1 score reached **99.10%** performance.

The algorithm always finds more cancerous parts than the ground truth regions, which is good since the annotations just cover the surely tumor and surely healthy parts; this could be measured as a qualitative evaluation made by the pathologists who annotated these regions. The experts confirmed (Rule 2) that the algorithm was also accurate by finding every tumor part with a small occurrence of misclassifying healthy parts.

## 4. Discussion

There are multiple applied ways to examine breast tissues, including Mammography, Ultrasound, Computer Tomography (CT), Magnetic Resonance Imaging (MRI) and Nuclear Imaging. In recent years, lots of articles have been published about computer-based techniques in order to segment and classify tissue images. Among women, the most common incident site of cancer was the breast, and due to the increasing number of patients and the small number of pathologists, an automated computer-aided diagnosis could help this complex and time-consuming procedure [4]. In computer-aided decision approaches, information technology is applied to help doctors to examine individuals [5]. There is a recent study [6] that reflects on the importance of assisting radiologists and healthcare professionals in the breast cancer classification process by introducing a new Ensemble Deep-Learning-Enabled Clinical Decision Support System using ultrasound images [7]. This research [6] was designed to identify the tumor-affected regions by multi-level thresholding-based image segmentation. MRI has also become a broadly used imaging technique that avoids ionization radiation and, therefore, it may be suitable for patients with implants [8]. It can also capture the structure’s higher gentle tissue resolution [8].

Although ultrasound and MRI provide non-invasive approaches, most researchers utilize the BreaKHis database [9] for their studies [8,10,11,12,13,14,15,16,17,18], which contains 7909 breast cancer images in RGB format with dimensions of 700 × 460 pixels in four different magnifications (40×, 100×, 200× and 400×). This database has been built in collaboration with the P&D Laboratory—Pathological Anatomy and Cytopathology [9]—including H&E stained microscopic breast tumor tissue image patches, which were annotated by experts. Most of the researchers split the database into training and test subsets, sometimes also using cross-validation samples [15]. Besides BreaKHis, other images were also applied. All of these breast cancer databases have similar characteristics, for example, using the Wisconsin Original Dataset [10], the University of Michigan and University of British Columbia Virtual Slidebox [19] or other individual databases [20,21,22]. The different magnifications of images and the multi-resolution databases are essential to make the models more robust and generalizable [23].

Our rule-based method provides a general approach to any segmentation problems presented on our own individual H&A stained microscopic dataset. The high-resolution images of this database were annotated to our special request of being more detailed than they usually are. All the separated cancerous regions were marked independently (Rule 1) instead of simply drawing a border between the tumor and the normal tissues. This area-based approach is unique because these annotations are time-consuming for the experts and expensive for the research institutes, but we consider that this algorithm will make decisions in a more accurate way of the not marked regions by learning the features only of the tumor and healthy samples without less noise, human error and redundant data. Our database was examined by histopathologists who were also involved in every stage of the implementation and validation processes (Rule 2). The database was split into training, validation and test sets containing multi-resolution images (Rule 3) to learn the features more robustly by a convolutional neural network.

Most publications also presented a convolutional neural network-based deep learning classification, which provides a large number of binary classifications, although a small number of papers used multi-class separation [13,24]. Detecting both, the subclasses of the benign and malignant tissues were 10% less accurate in comparison with the binary decisions [15], which are more important for computer-aided diagnosis to save time and help the pathologist to examine relevant areas for further analysis.

The algorithms were quantitatively evaluated on test patches. They were shown to reach 80–100% accuracy scores. The highest prediction accuracy achieved was 99.86% by a three convolutional and max pooling layered neural network [10]. The algorithm—which is magnification independent—is also provided by a convolutional neural network. This architecture was trained to predict the benign/malignant decision and the magnification factor by softmax loss minimization [11].

In the paper [25], the highlight is the collection of features, which are important in the classification process to minimize computation time and data size and increase the precision and effectiveness of machine learning approaches. Beyond classification, paper [8] also concerns approaches combining the outcomes as a segmentation result by sliding through the whole image. The paper introduces a deep neural network-based binary classification, evaluated on the ROIs of the slides with 50% overlapping steps, achieving 96.70% test accuracy.

Although we all know that the duration of the tissue sample evaluation is essential, only a few publications share relevant information with us about these issues. A U-NET-based epidermal tissue segmentation approach is executed on whole-slide histopathological images (17,111 × 17,145) in 3 min on average [19], and the Panoptes network predicts endometrial cancer by analyzing a slide with an appropriate GPU within 4 min [23] (Table 4).

The evaluation of our method used a sliding window technique to binary classify the tumor and healthy tissues at each pixel. The validation dataset considers the time and accuracy of the algorithm by displaying which parameter values are the best options to provide results as fast and also as accurately as possible (Rule 4). The algorithm was tested on the test dataset, reaching a 99.10% f1 score on pixel-level evaluation of a high-resolution image, averagely within 129 s.

## 5. Conclusions

The algorithms were evaluated by scikit-learn metrics machine learning library and manually by pathologists. The kernel level and the pixel level evaluation show us that these methods are accurate and quick enough to be an efficient alternative for a computer-aided diagnosis. The algorithms work on downsampled WSIs; thus, analyzing samples at different magnifications could be executed after reducing their resolution to the same level as the trained database, which is robust to small changes in magnification. In this paper, we present an individual approach to segment breast tumor tissues. We built our neural network model on an area-based, annotated (Rule 1) dataset where the tumor and healthy regions were precisely marked without any intermediate parts in order to avoid human errors. The experts—who annotated the dataset—were involved in the whole research process (Rule 2), especially at the preprocessing and evaluation stages. As we trained the convolutional neural network on a multi-resolution (Rule 3) dataset to learn the features most robust to noise and magnitude changes, we reached a 99.21% accurate model. The model was trained only on the tumor and healthy parts, but it was also able to segment any other regions of the image. The algorithm analyses the whole slide images by evaluating patches with a sliding window technique, which was optimized to speed up the segmentation process without decreasing its performance (Rule 4). The final segmentation parameters were set according to the trade-off between the evaluation time and the accuracy of the segmentation.

The qualitative evaluation of the experts (Rule 2) confirmed that the segmentation was also accurate even on regions not annotated before. In the future, developing a program for pathologists and enabling them to modify the predicted tumor areas by adding and extracting regions can be time-efficient and precise assistance for computer-aided diagnosis. This study was compared with other approaches: on kernel level evaluation with classification networks and on pixel level evaluation with segmentation results, considering the time factors as well. According to our experiments on the test dataset, this approach provides more accurate performance in less time, by reaching a 99.10% f1 score with a 3 min evaluation time on average.

Following the implemented rules helps to segment any other tumor tissue samples (e.g., ovarian) where the segmentation is even more complicated and time-consuming for pathologists. Applying the guidelines of this paper could be a straightforward solution to other modalities of imaging as well. According to our expectations, these methods will perform as accurately as on breast tumor tissue samples.

## Figures and Tables

**Table 1:** Database information.

Dataset	Train	Validation	Test
**Distribution**	70%	10%	20%
**No. of high-resolution samples**	201	29	61
**No. of tumor tissue tiles**	77,811	18,469	18,973
**No. of normal tissue tiles**	128,363	12,458	17,971
**Total no. of tiles**	206,174	30,927	36,944

**Table 2:** Average evaluation performances and times at different step sizes on the threshold level of 0.13.

Step Size	Accuracy	F1 Score	Evaluation Time (min)
128	96.99% ±3.36%	98.41% ±1.79%	0.13
64	98.17% ±2.59%	99.03% ±1.38%	0.53
32	98.26% ±2.50%	99.08% ±1.33%	2.15
16	98.32% ±2.38%	99.12% ±1.13%	8.63
8	98.34% ±2.33%	99.13% ±1.12%	34.52

**Table 3:** Kernel level test metrics with the final threshold.

Metric	Result
**Accuracy**	98.80%
**AUROC**	98.80%
**Precision**	98.81%
**Recall**	98.78%
**F1 score**	98.80%
**Mean squared error**	1.38%
**True Positive Region**	98.81%
**False Positive Region**	1.19%
**False Negative Region**	1.22%
**True Negative Region**	98.78%

**Table 4:** Comparing our approach to the other WSI segmentation techniques that consider the time factor as well.

Work	[19]	[23]	Our Work
**Model**	U-net	Panoptes	CNN
**Database**	69 skin	496 endometrial	291 breast
	WSI	carcinoma WSI	WSI
**F1 score**	89%	NA	99.10%
**AUROC**	NA	96.90%	98.80%
**WSI size**	17,111 × 17,145	NA	29,314 × 17,432
**Evaluation time**	136.5 s	240 s	129 s

## Data Availability

The data are not publicly available because they still need to be protected, so they are not provided.
